# Meaning in life among Chinese undergraduate students in the post-epidemic period: A qualitative interview study

**DOI:** 10.3389/fpsyt.2022.1030148

**Published:** 2022-11-04

**Authors:** Ying Ge, Cun Yang Lu, Mei Qiong Shuai, Jay L. Wenger, Chun Hua Peng, Hong Wang

**Affiliations:** ^1^Key Laboratory of Emotion and Mental Health in Chongqing, Chongqing University of Arts and Sciences, Chongqing, China; ^2^Social Sciences Division, Harrisburg Area Community College, Lancaster, PA, United States; ^3^Teaching Affairs Department, Chongqing Normal University, Chongqing, China

**Keywords:** post-epidemic period, Chinese undergraduate student, meaning in life, qualitative interview, COVID-19 epidemic

## Abstract

**Background:**

COVID-19 epidemic has lasted for nearly 3 years, and revolutionized social life. In the study, in-depth interviews were conducted with Chinese undergraduate students to explore their understanding and experience of meaning in life. Meaning of life is interpreted from four aspects: life goals, life value, life enthusiasm, and life freedom. These four aspects are independent yet interrelated. Based on the free grasp of life, individuals explore and pursue the true meaning of life goals, acquire life value in evaluating the completion of life goals, and subsequently experience enthusiasm for life. Life enthusiasm and the perception of life value can help individuals to further understand and possess their meaning of life.

**Materials and methods:**

The present study adopts the qualitative method to understand the experience of meaning in life among Chinese undergraduate students. Semi-structured interviews were conducted, and six people participated the study. The Grounded Theory was adopted to analyze the qualitative data.

**Results:**

(1) Chinese undergraduates had clear life goals and obtained a certain sense of achievement and satisfaction when striving for these goals. (2) The life value of Chinese undergraduates was mainly to their families, but there was also a willingness to make due contributions to the country and society. (3) Chinese undergraduates’ feelings about life were polarized, but they all expressed the view of “living in the moment and cherishing the present.” (4) Chinese undergraduate students see life freedom as freedom of choice and generally believed that COVID-19 did not restrict their lives very much. (5) Chinese undergraduate students gained a deeper understanding of meaning in life after this major public health emergency.

## Introduction

The outbreak of COVID-19 in early 2020 disrupted people’s lives (1). To combat the epidemic, countries around the world took actions that were effective to varying degrees. As new negative developments occurred in some countries, we stepped-up vaccination efforts to bring the epidemic under control. During this special period, Chinese undergraduate students experienced changes in their living environments and lifestyles—changes such as school closures, mask-wearing, nucleic acid testing, and health codes. These changes exposed many undergraduates to anxiety, fear, loneliness, depression, confusion, and helplessness (2–4). What is meaning in life for Chinese undergraduates facing the epidemic? What actions have they taken accordingly? Are there any special manifestations or patterns in their behavior? To answer these questions, this study combined semi-structured interviews and qualitative analyses for the real reflections of life meaning for Chinese students and the impact of the epidemic environment on life meaning.

Meaning in life was initially proposed by the famous psychologist Frankl (5). Subsequently, Crumbaugh and Maholic (6) figured that meaning in life carries a sense of existence and meaning, and that people perceive their value in the process of pursuing meaning in life. Reker (7) considered life meaning as a multi-dimensional structure consisting of a sense of continuity and satisfaction while striving for life goals. Wong and Fry (8) argued that life meaning includes cognition, motivation, and emotion. According to Steger et al. (9), life meaning consists of two dimensions, search for meaning and presence of meaning, and it represents an individual’s perception of self-value and meaning of existence. In the view of Song (10), life meaning is the goal guiding individuals to a sense of existence and value. Addressing the same topic, Li (11) divided life meaning into three parts: search for life meaning, self-transcendence, and life control. In general, meaning in life refers to the goal that directs individuals to their pursuit and value (12, 13). Meaning in life cannot only give people a sense of purpose and direction in life (14), but it can also generate self-value, a positive sense of meaning, and be a driver for upward development for the individual (9).

As a result of COVID-19, people’s mental health has been affected in multiple ways, especially in terms of anxiety, depression, interpersonal relationships, and life satisfaction. Emotional problems, in particular, are most prominent when people face a public health emergency (15). In the early stage of the epidemic, such problems stemmed from the degrees to which people could sense meaning in their lives (16). However, as the epidemic gradually subsided, people’s negative psychological symptoms also subsided as they gained more understanding of the epidemic through the media and were able to adjust their cognitive strategies (17).

Undergraduates are in the early stage of youth, a critical period for ideological and personal development (18, 19). They have the will to search for life meaning (20), but they can be easily affected by the external environment and their confusion about growth, which often leads to a lack of meaning in life (21, 22). Thus, an undergraduate’s life meaning is closely related to their mental health (19, 23, 24). Those with higher levels of life meaning enjoy greater optimism (25–27), better interpersonal communication (28–30), more positive value experience (31, 32), more happiness in life (33), more hope and satisfaction with life (34, 35), and healthier mental states (36, 37). Furthermore, search for meaning and presence of meaning can keep people away from negative mental states, such as depression (37), suicide (21), self-identity crisis (38), and loneliness (39). In contrast, those with lower levels of life meaning may suffer from a series of psychological problems, including a decline in interest and motivation (22), reduced perception of social support (33), addiction (40), emptiness, depression, and anxiety (33).

Previous research has established that only when individuals have a clear understanding of their own life experiences and a clear sense of purpose and direction in life through the interpretation of these experiences, then they can gradually acquire the ability to give meaning to their lives (9). The acquisition of meaning in life originates from the individual’s active pursuit and construction (41). Cognitive appraisal is an effective adaptation mechanism when facing a stressor. Throughout the extent of COVID-19, the cognitive appraisal of undergraduate students changed. At the beginning, there was uncertainty, confusion, and a lack of understanding. So, it was difficult to cope with this new stressful event. Eventually, however, students were able to reduce adverse psychological reactions and actively pursue and construct meaning in life during the recovery period of COVID-19 (42).

According to Frankl (5), meaning in life is at the core of human happiness. One study has shown that collectivism is significantly and positively correlated with life satisfaction and emotional well-being. China is saturated with a collectivist culture (43). Thus, research related to meaning in life is paramount for people in a country like China.

To sum up, undergraduates have the will to pursue meaning in life, but their understanding and grasp of life meaning are subject to the external environment and their confusion about growth. Understanding life meaning is an essential part of undergraduates’ mental growth. What characteristics and patterns have Chinese undergraduates shown, especially during a unique time like the COVID pandemic? This is an important question that deserves in-depth analysis. Ge et al. (44) used a quantitative study to evaluate meaning and well-being among Chinese undergraduates. But reducing complex mental health variables to quantitative data can be overly simplistic (45). Thus, Kelle (45) argues that it is important to supplement quantitative findings with qualitative research.

Therefore, the current study adopted a qualitative approach of deep interviews based on the Grounded Theory to explore Chinese undergraduates’ understanding and experience of life meaning in the post-epidemic period, aiming to facilitate corresponding research and provide a better reference for monitoring undergraduates’ mental health and promoting life education.

## Materials and methods

### Participants

Sampling for qualitative research usually follows the non-probability principle, and purposive sampling is very common (46). Thus, we used purposive sampling, with all participants recruited online but interviewed offline. The following factors were considered in recruitment: (1) the ratio of male to female was set 1:1 to control for gender; (2) middle-grade (sophomore and junior) undergraduates were selected to avoid the influence of insufficient life experience or upcoming graduation; (3) ages 18–22 were selected; (4) a balance between arts and sciences was taken into account. Furthermore, the participants were required to be fluent in verbal communication and have no major mental disorders (see Table 1).

**TABLE 1:** Basic information of participants.

Subject No.	Gender	Age	Grade	Major
01	Female	18	Grade 2019	Cultural heritage
02	Male	21	Grade 2018	History
03	Female	22	Grade 2018	Elementary education (Teaching)
04	Male	21	Grade 2019	Electronic information
05	Male	18	Grade 2019	Electronic information
06	Female	20	Grade 2018	Preschool education (Counterpart)

### Method

#### Interview outline

To better inspire participants and guide them to reflect on their life meaning, we used semi-structured, in-depth interviews. The questionnaire outline is constructed with reference to Dong’s (13) revised version of the *Meaning in Life Questionnaire* (MLQ), originally proposed by Song (10). It consists of four dimensions: life goal (degree of control over life goals), life value (identification with one’s value), life enthusiasm (feeling about one’s current life), and life freedom (autonomy of one’s life). MLQ is derived from the *Purpose in Life Test* (PIL), developed by Crumbaugh and Maholick (47) and based on Frankl’s logotherapy theory. Repeatedly revised and used, the MLQ is applicable in Chinese contexts (10, 13, 48–51). Simultaneously, it is comprehensive and conducive to a deeper exploration of individual views and understanding of various aspects of life meaning. The open-ended question outline designed against the MLQ structure is as follows (Table 2).

**TABLE 2:** Interview outline on Chinese undergraduates’ life meaning in the post-epidemic period.

Dimensions	Questions
Life goal	1. What do goals mean to you? What are your short-term and long-term goals for all aspects of life and study? 2. What efforts have you made to achieve these goals? Have you achieved your goals through these efforts? (Feelings about success/failure) 3. Did your goals change during the epidemic? (Feelings/specific changes)
Life value	1. Please make an evaluation of your own value as detailed as possible. (What kind of role are you playing with yourself and others, or in a group? What value have you acquired?) 2. What is your understanding of life value? (Value of your own life/others’ lives; Did your opinions change during the epidemic?)
Life enthusiasm	1. How would you describe your current life and how do you feel about it? (Why do you have such feelings? Please give more details.) 2. Can you identify ways to improve your current life, increase your happiness, and maintain your enthusiasm for life? What attempts have you made to this end? What results have you achieved? (Feelings?) 3. Is there any difference between your feelings about life now and during the epidemic? Has your life enthusiasm changed? (Please describe in detail.)
Life freedom	1. How do you perceive the “freedom of choose”? What do you think of it? (What is your understanding of freedom?) 2. Was your life affected greatly under strict travel restrictions? (Any sense of restraint in family contact, shopping, communications with friends, classmate reunion, etc.? Or any other feelings?)

#### Interview setting

In-depth interviews were conducted face-to-face and one-by-one in a quiet and comfortable room. Each interview was 50–60 min long. The interviewers were professional psychology researchers who remained neutral throughout the study so participants felt accepted and not judged. After participants signed informed consents, the researchers began recording and interviewing them. At the end of each interview, the researcher thanked the participant with a small gift. The study was approved by the Ethics Review Committee at the primary researchers’ university.

#### Data acquisition and analysis

Audio recordings from the participants were transcribed into text that totaled 12,569 words and analyzed through the N-Vivo 11.0 software and based on the grounded theory. Glaser and Strauss (52) proposed the grounded theory, which was initially applied to the field of sociology and later used for a variety of topics in psychology, including environmental psychology, health psychology, clinical psychology, and psychotherapy (53).

The grounded theory approach is based on the systematic collection of information to find the core concepts that reflect social phenomena, construct connections between concepts, and eventually develop a theory (54). This approach advocates paying attention to all the details in the process of collecting materials in order to make them rich and exhaustive. The analysis of materials is achieved by categorizing them in a hierarchical coding system. According to the degree of abstraction, the coding can be divided into three different levels: primary coding is open coding, which requires the researcher to objectively code all materials in their original state; secondary coding is axial coding, which is used to establish the relationship between concepts and explore the internal connection of each part of the material; tertiary coding is selective coding, which establishes the core category and concentrates related concepts in the core category (55, 56).

In this study, meaning of life is interpreted from four aspects: life goals, life value, life enthusiasm, and life freedom. These four aspects are independent yet interrelated. Based on the free grasp of life, individuals explore and pursue the true meaning of life goals, acquire life value in evaluating the completion of life goals, and subsequently experience enthusiasm for life. Life enthusiasm and the perception of life value can help individuals to further understand and possess their meaning of life. Thus, the current research focuses on core coding through semi-structured interviews, sort-out and code the relationship between each node, and outline the interview content to each core category to elaborate new connotations (55, 56; Table 3).

**TABLE 3:** The relationship and distribution of each coding node.

Core coding	Axial coding	Total number of reference points
Life goals	Goal recognition, goal practice, epidemic impact	35
Life value	Value recognition, value practice, epidemic impact	44
Life enthusiasm	Status perception, improvement approach, epidemic impact	30
Life freedom	Viewpoints and opinions, freedom recognition, epidemic impact	28

As stated, audio recordings from the participants were transcribed into text that totaled 12,569 words. The specific steps were: (1) getting immersed in the text of each case by repeated reading; (2) identifying meaningful text, generating initial open codes, and writing notes, reflections, and comments; (3) thinking about the open codes and all the notes, reflections, and comments, until these are summarized appropriately, and classified into different core concept categories; (4) turning to the next case and repeating the above steps for all cases.

## Results

### Life goals

**FIGURE 1:**
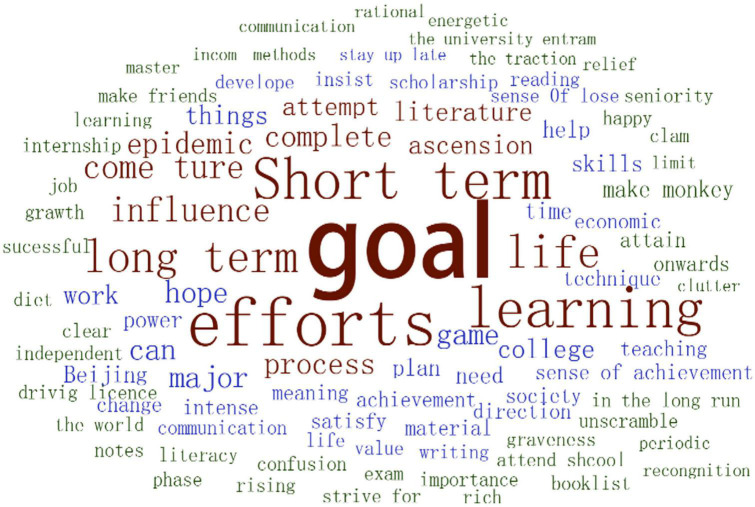
Word cloud about life goal.

According to the word cloud (Figure 1), the goals mentioned by the participants concern two aspects: life and study. They emphasized future needs and their own interest when setting goals, and focused on skill development and mental growth when striving for these goals.

After sorting the interview materials, it was found that Chinese undergraduate students have relatively clear life goals (Table 4). These students generally agreed that goals and motivation (pulling force) were important factors in making life meaningful. Things were different for each participant, but there were also similarities: After a time of consideration, most participants could figure out their short-term and long-term goals quite fluently and list their efforts to achieve these goals. In this goal-pursuing process, there was positive feedback, like increased confidence and a sense of achievement. If the goals were not accomplished, the participants would feel discouraged, but eventually re-adapt themselves. The epidemic has not had a major impact on participants’ goals, but rather clarified their goals and strengthened confidence for executing their plans.

**TABLE 4:** Long-term and short-term goals.

Subject No.	Major	Short-term goals	Long-term goals
1	Cultural heritage	To pass Chinese CET 4 (College English Test 4) To get a driving license To win a scholarship To learn how to wear make-up	To pass the postgraduate exam (further study in Beijing Foreign Studies University) To obtain a teacher qualification certificate in China To learn how to manage finances To stay in Beijing for work
2	History	To learn about various regions of the world in the 7th century B.C. To eat, read, and socialize normally	To enroll in a Ph.D. program To settle in the eastern coast of China
3	Elementary education (Teaching)	To find a good job To achieve financial independence To pass the final exam	To be self-sufficient in life To become a teaching teacher To improve literary attainment To be a moral man
4	Electronic information	To increase incomes To win a scholarship	To further study in Jiangsu, China To live for myself
5	Electronic information	To improve game skills To pass exams and complete assignments To eat well, and hang out with extra money	To have a girlfriend
6	Preschool education (Teaching)	Not to fail the major exams To read the professional books I like and do mind mapping To read recommended books if there is more time To make full use of time through time management	To become an educated and cultured postgraduate To buy a house To support myself as a freelancer To be successful to a degree in my favorite field

### Life value

**FIGURE 2:**
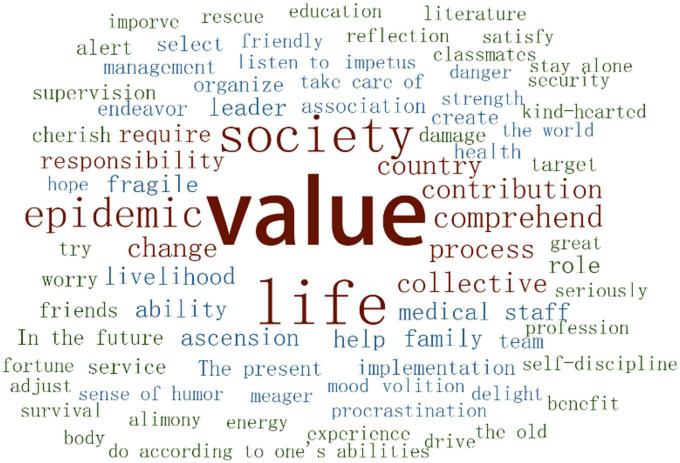
Word cloud about life value.

According to the word cloud, undergraduate students combine self-value and social value in their understanding of life value (Figure 2). To realize life value, they need to make plans and generate sufficient motivation under the impact of the external environment. For many participants, role models inspired them during the epidemic to view life meaning and value in a new way: their life value will not be fully achieved unless they satisfy the needs of both self-growths along with social and national development.

For the most part, participants linked their life value with the concepts of society, family, school, and group, and they evaluated their value based on their contributions to other people, the collective, and society. Most participants can feel their own value when getting along with others. It was reflective of self-value to contribute to a group, to shoulder responsibilities in the family, and also to be self-disciplined and self-guided. However, there was also a majority of participants who gave social value a relatively low score on account of limited competence to contribute direct value to society (Table 5).

**TABLE 5:** Response examples about life value.

Categories	Subject No.	
	2	6
Value cognition	Life itself has no value. Life value is a choice and a subjective judgment, and it depends on you to decide your maximum life value. Likewise, others’ value lies in their own choice.	Life value represents an individual’s understanding of how to live and what life goal he or she needs to achieve. Life value cannot be measured by anyone.
Value practice	It means to be my own guider and enabler, to be humorous and sensitive to emotions when getting along with others, and to be an ice breaker in a group.	It is required to accept my shortcomings and be brave and kind; to take good care of grandparents and parents in the near future; and to study hard, benefiting society with my knowledge.
Epidemic impact	Medical workers’ life value was enhanced during the epidemic when they saved more lives.	Life is fragile against nature. We must enhance vigilance, take precautions, and cherish life.

Generally, participants believed that life value lied in creating value for society and others; it was a choice of their own that cannot be measured by others. To realize self-value, one must do what he or she should do at the moment. Thanks to the epidemic, participants refreshed their understanding of life value and improved it by valuing the moment and living a positive life. Furthermore, they became convinced that those who contributed to society during the hard times were enjoying higher life value. This emphasizes the importance of becoming useful to society and country for self-value enhancement (Table 5).

### Life enthusiasm

**FIGURE 3:**
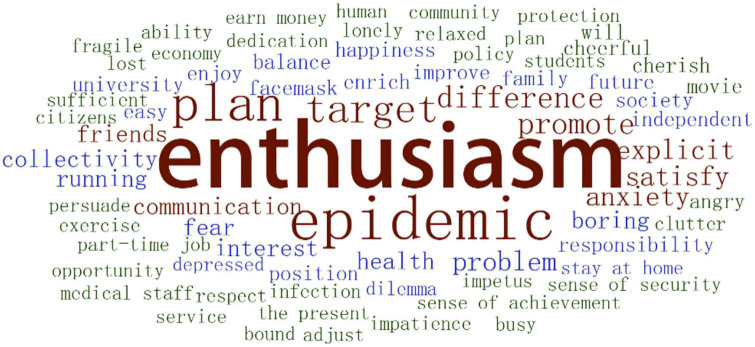
Word cloud about life enthusiasm.

In terms of life enthusiasm, the word cloud showed that undergraduate students felt bored staying at home under travel restrictions during the epidemic. But they neither changed their goals nor developed strong anxiety and uneasiness. On the whole, they were still enthusiastic about life because they were convinced that the epidemic was controllable, and that society and modern medicine were trustworthy. Feelings about current life were different from those during the home segregation as negativity decreased. There was more emphasis to cherish the hard-earned life and live in the moment (Figure 3).

Some participants complained about current boredom, unnecessary busyness, and unbalanced and unclear schedules, while the others were satisfied that life was full and on the right path as they have planned. Going through the epidemic, they identified specific ways to better appreciate the moment. Some participants reported clear plans to improve their life experience and were repaid with a greater sense of achievement, happiness, and enthusiasm (Table 6).

**TABLE 6:** Response examples about life enthusiasm.

Categories	Subject No.	
	2	4
Current feelings	I’m well-clothed, well-fed, and healthy. Life is advancing as I have planned. But I will feel angry when my plan is interrupted because I don’t like changes.	I feel bored, lonely, and helpless when I cannot get along well with my friends and have to lock myself in my own world. But sometimes, I enjoy such solitude.
Adaptation methods	I know how I should change myself, and I’m trying. I’m making my life less dogmatic, learning to deal with changes, and allowing myself more relaxation in a month. This feels good and comfortable.	I’ve identified ways to adapt myself, and I’m trying to make a difference. I do sports on the playground myself, which is a good way to relieve my stress.
Epidemic impact	I see no difference, because life plans and needs remain the same and have to be met as usual.	Differences do exist. Life during the epidemic was depressing and constraining, but it’s much better now. I can do what I want to do.

The epidemic has impacted Chinese undergraduates in transportation and communication. But as undergraduates gradually adapted to these changes, they enjoyed more freedom, fun, and enthusiasm under the additional rules. In other words, they were less anxious or depressed (Table 6).

### Life freedom

**FIGURE 4:**
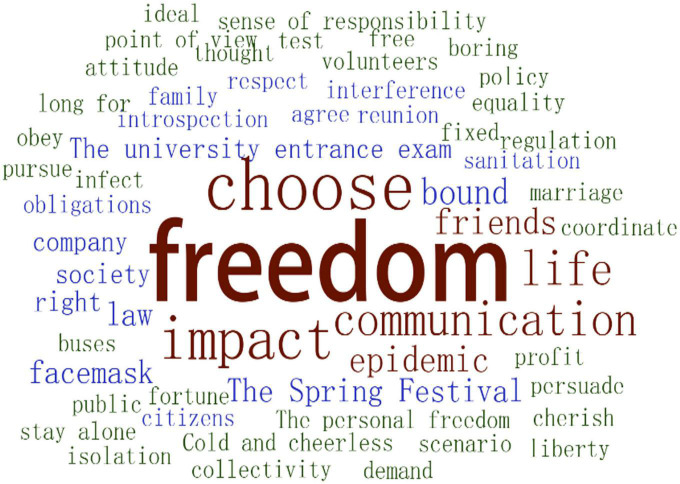
Word cloud about life freedom.

As the word cloud shows, Chinese undergraduates believed that freedom meant the ability to make choices within certain limits and to take responsibility for their choices (Figure 4).

In the view of undergraduate students, freedom was relative within the confines of law and morality. There were some things that were beyond their subjective choices. The freedom of choice also suggested that they could make choices with a will of their own and be responsible for those choices. They could choose to avoid the influence of others, respect everyone’s right of choice, and still remain true to themselves. But everything was subject to law and moral requirements: choices and decisions should not harm the interests of the country, society, or others (Table 7).

**TABLE 7:** Response examples about life freedom.

Categories	Subject No.	
	3	5
Freedom cognition	Freedom means doing what I like under relative conditions.	There is relative freedom when you obey rules under the constraints of the law.
Opinions on the freedom of choice	I agree that people have the freedom of choice, but it depends on the specific situation and perspective. To be more accurate, people have the relative freedom of choice.	Life is like an exam; you don’t know the right answers. However, you can make all the choices and be responsible for them.
Epidemic impact	I felt no restraints. As a citizen, I have the responsibility to be in line with epidemic prevention. When I volunteered and was isolated during the epidemic, I reflected on my contact with my parents and treasured my family and friends more than ever.	I couldn’t gather with my classmates, but I could communicate with them online. Life went on as usual, to some extent. One thing worth celebrating was the closer bond between my parents and me.

The majority of participants felt restricted in travel during the epidemic, especially when taking public vehicles, which required safeguards, and when offline communication was unavailable. But such restrictions also allowed them to enjoy more solitude and encouraged more contact with friends and family. Some participants thought they increased communication with their parents. In short, the epidemic has not posed a long-term impact on undergraduates’ sense of freedom and well-being (Table 7).

## Discussion

In this research, Chinese undergraduate students had relatively clear life goals, and they desired to accomplish the goals through their own efforts—a process where they obtained positive experiences. The epidemic did not have much of an impact on their life goals but rather led to an increased sense of control over the goals. This is probably because, when a major public health emergency occurs, an individual’s mental state will change with the external environment (57). Since such an impact can last for a long time, it will likely act on undergraduates’ life goals even in the phase of prevention and control (58).

Undergraduate students found their life value mainly from their families and friends and were disappointed that they could not contribute more to their country and society. The epidemic apparently taught them to view their social value from a new angle. They are now active in pursuing self-growth along with the development of the country and society. They expect themselves to contribute more to the country and society and truly realize self-value through hard work at college. This is consistent with previous findings that undergraduate students emphasize life responsibilities and desire to serve the country and society (59, 60).

In terms of life enthusiasm, undergraduates’ feelings about life were polarized: extreme boredom compared to satisfying fullness. In general, they all emphasized one thing, and that was to live in the moment and cherish the present. According to the results of our interviews, the special experience about life and death has enabled undergraduates to have a deeper understanding of life value. This coincides with the Chinese culture’s optimistic interpretation of difficulties: “How long can life be? Full admiration is that dreamy beauty worth.” “Fret not over bygones and the forward journey take” (61).

Unlike the polarized view for life enthusiasm, undergraduates shared similar opinions on life freedom, although freedom was relative under the constraints of the law. They agreed that “people have the freedom of choice” and need to be responsible for their choices. Despite travel restrictions, the epidemic has deepened the relationship between undergraduate students and their parents. It has also increased time for solitude, thus minimizing the sense of restraint. Life freedom refers to the autonomy of an individual’s life. As young adults, undergraduates are relatively independent and able to run their own lives (18). Therefore, when COVID-19 broke out, these autonomous individuals could deal with it fairly well and take good care of themselves.

In his work *Man’s Search for Meaning*, Frankl (5) mentions three important ways to search for life meaning: to do meaningful things, to love oneself and others, and to have the courage to overcome difficulties. In the phase of regular prevention and control, life remains an uncertainty. However, having gone through the tough pandemic, undergraduate students have refreshed their understanding of meaning in life. They are moving ahead steadily as they strive for goals, enjoy life, pursue freedom, and search for value. By and large, they are living in the moment and cherishing the present.

According to Battista and Almond (62), the development of meaning in life is divided into two stages: positive ego orientation and positive life orientation. The second stage occurs during late adolescence and early adulthood, where the main task of the individual is to develop a positive life concept and become aware of the meaning in life. This coincides with the contemporary state of undergraduate students, because they pursue goals, along with enjoyment of life, and values in life. In addition, Steger et al. (63) found that younger age groups were more motivated to pursue meaning in life through studying corresponding age trends. Undergraduates in the post-epidemic period actively seek to construct meaning in their lives by refining life goals, experiencing life values, and using cognitive strategies to improve the integration of their interactions with the environment.

Undergraduates are in a positive life orientation phase, and existential positive psychology emphasizes that such positive orientation should be accompanied by embracing the negative aspects of life (9). In a life course where good times and bad times coexist, people should construct and create meaning from negative scenarios (41) and transcend real-life dilemmas. This is very consistent with the performance of undergraduate students in the current study. Although participants experienced anxiety, panic, and limitations during the epidemic, they were committed to finding new perspectives from which to create meaning from these negative events.

In conclusion, Chinese undergraduate students have a unique understanding for meaning of life. The anxiety, depression, and fear they had during the epidemic were not insurmountable. As China enters the phase of regular epidemic prevention and control, undergraduates’ negative emotions are fading as they realize they need to co-exist with viruses for a long time. They are blessed with rising adaptability and mental toughness, showing their confidence in modern medicine and technologies. In spite of its negative impacts, the epidemic has reawakened undergraduate students to reflect on life, and to be clearer about life goals and meaning. This has further inspired them to re-examine themselves, which is, in some sense, “a new stage of enlightenment” (64).

## Conclusion

(1) Chinese undergraduates in this study have clear life goals and obtained a certain sense of achievement and satisfaction when striving for these goals.
(2) The life value of Chinese undergraduates in this study is mainly tied to their families, but there is also a willingness to make due contributions to their country and society.
(3) In this research, Chinese undergraduates’ feelings about life are polarized, but they all express the importance of “living in the moment and cherishing the present.”
(4) Chinese undergraduates in this study see life freedom as relative freedom of choice, and they generally believe that COVID-19 has not restricted their lives very much.
(5) Chinese undergraduates in this study gained a deeper understanding of life meaning after this major public health emergency.

## Enlightenment and prospect

(1) The pursuit of life’s purpose is an important way to gain meaning in life. The epidemic has enhanced the appreciation and reverence for life. After experiencing a major stressful event, we can guide undergraduates to sort out their correct life goals and promote their mental health by strengthening their life awareness.
(2) The pursuit of social values by the undergraduates in this study suggests that fostering their sense of altruism and responsibility can enhance their sense of meaning in life and thus promote their mental health.
(3) Lazarus believes in emotions as a response to meaning, which is determined and accomplished through cognitive evaluation (65). Therefore, in our educational activities, we can consciously foster the application of positive cognitive strategies in undergraduate students to motivate them to actively create, construct, and pursue meaning in their lives.

## Limitation and future research

To the best of our knowledge, this study is the first attempt to reveal the understanding and experience of meaning in life among Chinese undergraduate students in the Post-epidemic Period. The six cases of this study are from one single university in Southwest China. Thus, due to the small sample size, the findings may be region-specific, and generalizability might be limited. Additional studies should examine and replicate our findings in other regions in China, along with other areas with different cultural contexts—exploring the understanding and pursuit of undergraduate students on the meaning in life. Moreover, in order to implement a more universal and extensive life understanding, future research with other youth groups is needed—for example, adolescents and young employees.

## Data availability statement

The raw data supporting the conclusions of this article will be made available by the authors, without undue reservation.

## Ethics statement

The studies involving human participants were reviewed and approved by Chongqing University of Arts and Sciences Institutional Review Board. The participants provided their written informed consent to participate in this study. Written informed consent was obtained from the individual(s) for the publication of any potentially identifiable images or data included in this article.

## Author contributions

YG, CL, and JW contributed to the study conception and design and wrote the first draft of the manuscript. CL, MS, and HW performed the interview and data collection. CL, MS, and CP were responsible for data collation, mining, and analysis. All authors contributed to the article and approved the submitted version.
